# *Rhizophagus irregularis* and *Rhizoctonia solani* Differentially Elicit Systemic Transcriptional Expression of Polyphenol Biosynthetic Pathways Genes in Sunflower

**DOI:** 10.3390/biom10030379

**Published:** 2020-03-01

**Authors:** Younes Rashad, Dalia Aseel, Saad Hammad, Amr Elkelish

**Affiliations:** 1 Plant Protection and Biomolecular Diagnosis Department, Arid Lands Cultivation Research Institute, City of Scientific Research and Technological Applications, New Borg El-Arab City 21934, Egypt; yrashad@srtacity.sci.eg (Y.R.); saadhmmad52@yahoo.com (S.H.); 2 Botany Department, Faculty of Science, Suez Canal University, Ismailia 41522, Egypt

**Keywords:** Arbuscular mycorrhizal fungi, chlorogenic acid, flavonoids, plant immunity, qRT-PCR, *Rhizoctonia* root rot

## Abstract

Plant roots are exposed to penetration by different biotrophic and necrotrophic fungi. However, plant immune responses vary, depending on the root-penetrating fungus. Using qRT-PCR, changes over time in the systemic transcriptional expression of the polyphenol biosynthesis-related genes were investigated in sunflower plants in response to colonization with *Rhizophagus irregularis* and/or infection with *Rhizoctonia solani*. The results demonstrated that both fungi systemically induced the transcriptional expression of most of the addressed genes at varying degrees. However, the inducing effect differed according to the treatment type, plant organ, targeted gene, and time stage. The inducing effect of *R. irregularis* was more prevalent than *R. solani* in the early stages. In general, the dual treatment showed a superior inducing effect over the single treatments at most of the time. The hierarchical clustering analysis showed that cinnamate-4-hydroxylase was the master expressed gene along the studied time period. The cell wall lignification was the main plant-defensive-mechanism induced. In addition, accumulations of chlorogenic acid, flavonoids, and anthocyanins were also triggered. Moreover, colonization with *R. irregularis* improved the plant growth and reduced the disease severity. We can conclude that the proactive, rather than curative, colonization with *R. irregularis* is of great importance, owing to their protective and growth-promoting roles, even if no infection occurred.

## 1. Introduction

*Helianthus annuus* L., which is widely known as a common sunflower, is among the most important oil crops in the world, including Egypt. Based on the global economic value of oil crops, it is ranked fourth after palm, soybean, and canola. The sunflower crop has a variety of food and industrial uses, such as oil production, livestock and poultry feeds, and the manufacturing of paints and cosmetics. In 2017, the global area under sunflower cultivation was 26,533,596 ha with a total production of 47,863,077 tons [1]. Nevertheless, the sunflower crop is exposed to infection with many destructive fungal pathogens [2,3].

*Rhizoctonia solani* J.G. Kühn 1858 (Family: Ceratobasidiaceae), which is the causal agent of Rhizoctonia root rot of sunflower, is one of the most destructive soil- and seed-borne phytopathogenic fungi, which infects a wide range of plant hosts resulting in high yield losses [3,4,5]. This necrotrophic pathogen produces sclerotia (overwintering structures), which can survive for many years in the soil or plant debris and then germinate, at the favorable conditions, to give mycelia, which attack the sunflower seedlings, causing necrotic lesions on the root and stem at the soil line (collar region) [6]. In addition to root rot, *R*. *solani* can cause various diseases on several plants, such as white blight, brown patch, aerial blight, sheath blight, damping-off, and target spot [7].

*Rhizophagus irregularis* (Błaszk., Wubet, Renker & Buscot) C. Walker & A. Schüßler 2010 (former *Glomus intraradices*) is an obligate biotrophic arbuscular mycorrhizal fungus that belongs to phylum: Mucoromycota, subphylum: Glomeromycotina, class: Glomeromycetes [8], and forms mutualistic symbioses with various plant species [9]. Several beneficial effects have been widely reported as a result of this relationship, including the enhancement of plant growth, nutrient and water transport, improvement of heavy metal, salinity, and drought tolerance, and the induction of plant disease resistance [10,11,12,13]. In this regard, El-Sharkawy et al. [14] reported a significant reduction in the stem rust of wheat, which is caused by *Puccinia graminis* Pers. f. sp. *tritici*, and a significant induction in defense-related enzymes and total phenol content of wheat plants when colonized with arbuscular mycorrhizal fungi under the greenhouse conditions. Various mechanisms have been discussed to be exerted by arbuscular mycorrhizal fungi against plant fungal pathogens, such as *R*. *solani* including the induction of the biosynthesis of many fungitoxic phenolic secondary metabolites [15]. Among these polyphenolic compounds, flavonoids and chlorogenic acid, which constitute the majority of polyphenols, have shown broad antifungal activities against several fungal phytopathogens [16,17]. Their antifungal mechanisms include fast membrane permeabilization in fungal spores and mycelia, DNA fragmentation, and mitochondrial dysfunction [18].

In sunflower, the phenolic compounds represent the most prominent bioactive compounds [19]. The polyphenol biosynthetic pathway can be divided into three sections: the main phenylpropanoid, flavonoid, and the chlorogenic acid biosynthetic pathway. The phenylpropanoid pathway start with the conversion of L-phenylalanine by phenylalanine ammonia-lyase (*PAL*) to cinnamic acid then to *p*-coumaric acid by cinnamic acid 4-hydroxylase (*C4H*)*,* and ended by the formation of the main intermediate coumaroyl-CoA that is controlled by 4-coumarate-CoA ligase (*4CL*). In the flavonoid pathway the coumaroyl-CoA can be converted by chalcone synthase (*CHS*) to chalcone, which can be converted to many intermediates flavonoids compounds by the action of enzymes set such as chalcone isomerase (*CHI*)*,* flavanone 3-hydroxylase (*F3H*)*,* flavonol synthase (*FLS*), flavonoid 3′ hydroxylase (*F3*′*H*), and dihydroflavonol 4-reductase (*DFR*). The flavonoid pathway is ended by the conversion of anthocyandin to anthocyanin and regulated by anthocyanin 1 transcription factor (*AN1*) and anthocyanin 2 transcription factor (*AN2*). In the chlorogenic acid biosynthetic pathway, coumaroyl-CoA can be converted into chlorogenic acid by the action of three main enzymes: hydroxycinnamoyl Co A quinate hydroxycinnamoyl transferase (*HQT*), hydroxycinnamoyl Co A shikimate hydroxycinnamoyl transferase (*HCT*), and *p*-coumarate 3-hydroxylase (*C3H*) [20,21,22]. In this study, two soil-borne fungi were addressed; both of them can penetrate and inhabit the plant roots, one behaves as a friend and the other as a foe. However, the plant might differentially respond to their penetrations. Here, we aimed to investigatie 1) the time-course changes in the systemic transcriptional expression of the flavonoid and chlorogenic acid biosynthetic pathways genes in sunflower plants, 2) the probable plant-defense-mechanisms, and 3) the plant health and growth development in response to root colonization of sunflower with *R. irregularis* and/or infection with *R. solani*.

## 2. Materials and Methods

### 2.1. Fungal Isolates and Sunflower Cultivar

A virulent isolate of the fungal pathogen *R. solani* AG-4 HG-I (PPR1745), which was isolated from sunflower seeds, was obtained from the Plant Patholology Reseach Institute, Giza, Egypt. A monosporic culture of *R. irregularis* propagated under sudangrass (75% colonization index) was used in this study. The utilized inoculum comprised rhizospheric soil and mycorrhizal root pieces. The seeds of sunflower cv. Giza 102, which were obtained from the Agricultural Research Center, Egypt, were used in this study.

### 2.2. The greenhouse Experiment

Four healthy sunflower seeds (cv. Giza 102) were firstly surface sterilized while using sodium hypochlorite solution (0.5%), before sowing in each pot. The plastic pots (15 Kg) are filled with clay-sand soil (1:2) and, at the sowing time, half of the utilized pots were inoculated with *R. irregularis* inoculum (50g pot^−1^) as a seedbed. No fertilization treatments were applied in this treatment. The pots were orderly irrigated with tap water to near field capacity. Thirty days after sowing, soil infestation was applied by mixing the *R. solani* inoculum with the upper layer of the soil at 2.5% (*w*/*w*). A set of pots only treated with sterilized tap water was used as a negative control. Seven pots for each treatment were used as replicates. The applied treatments were designated, as follows: untreated control (C), infected with *R. solani* (P), colonized with *R. irregularis* (M), and infected with *R. solani* and colonized with *R. irregularis* (M+P). The pots were arranged in a complete randomized design and kept under greenhouse conditions at 32/20 °C day/night and 74% relative humidity.

#### Time-Course Analysis of Gene Expression Changes

##### RNA Extraction and cDNA Synthesis

For the time-course analysis, sunflower leaves (2nd leaf from the base) of each treatment were sampled at different intervals (3, 7, 14, 21, and 28 dpi) and stored at −80 °C until mRNA extraction. While, root samples were sampled at 28 dpi. mRNA was extracted using RNeasy Mini Kit (Qiagen, Hilden, Germany) according to the manufacturer’s instructions.

First strand cDNA was synthesized from a reaction mixture (20 µL) containing 9.5 µL RNase free water, 0.5 µL MMLV reverse transcriptase enzyme (200 unit µL^−1^) (ABT H-minus cDNA synthesis kit, Applied Bioscience, Ismailia, Egypt), 1 µL oligo (dT) primer (10 pmol µL^−1^), 2 µL of dNTPs (10 mM), 3µL RNA (30 ng), and 4 µL of 5× first-strand buffer.

##### Quantitative Real-Time PCR (qRT-PCR)

The specific primer sequences of sunflower genes, *PAL1, C4H, CHS, CHI2, F3H, FLS1, DFR, F3′H, AN1, AN2, HCT, HQT,* and *C3H,* were designed and are mentioned in Table 1, and the *β*-actin gene was used as an internal standard due to their stable expression in sunflower based on previous studies [19]. The qRT-PCR reaction was performed while using a CFX Connect^TM^ Real Time System (BIO-RAD, Hercules, CA, USA). It was made up of 2 µL cDNA, 0.8 µL of each forward and reverse primers (10 pmol µL^−1^), 6.4 µL of RNase free water, and 10 μL 2xSYBR^®^ Green RT Mix (Bioloine, Luckenwalde, Germany). The PCR conditions were as follows, one cycle at 95 °C for 5 min., 40 cycles (95 °C for 5 s, 60 °C for 10 s, and 72 °C for 20 s). The relative expression of the tested genes was calculated in accordance to comparative C_T_ method (2^−ΔΔCT^) [23]. Triplicate biological and technical replications were applied for each treatment. The relative expression of the tested genes was expressed as log_2_-fold change respect to control to enhance the distribution of the data and the symmetry of the ratios.

### 2.3. Growth Parameters Evaluation

Twenty-eight days after inoculation with *R. solani*, four entire plants from each treatment were carefully uprooted, washed under running water, and evaluated for the shoot and root lengths (cm), shoot and root dry weights (g), and number of leaves, and leaf area. The dry weights were determined after drying the plant samples in an oven at 80 °C for 72 h.

### 2.4. Disease Assessment

Twenty-eight days after inoculation with *R. solani*, four plants of each treatment were evaluated for the disease incidence and severity. The disease incidence was calculated while using the Equations (1) and (2):

(1) $$\mathrm{Disease}\;\mathrm{incidence}\;\left(\%\right)=\frac{\mathrm{Number}\;\mathrm{of}\;\mathrm{infected}\;\mathrm{plants}}{\mathrm{Total}\;\mathrm{number}\;\mathrm{of}\;\mathrm{plants}\;}x\;100$$

Disease severity was estimated as the degree of root damage according to the scale of Carling et al. [24], as follows: 0 = no damage, 1 = minor discoloration of hypocotyl, 2 = discoloration plus small necrotic lesions (<1mm in diameter) on hypocotyl, 3 = discoloration with large necrotic lesions (≥1mm in diameter) on hypocotyl, and 4 = death of the plant.

(2) $$\mathrm{Disease}\;\mathrm{severity}=\frac{\sum \left(ab\right)\times 100}{AK}$$

where *a* = number of diseased plants having the same degree of infection, *b* = degree of infection, *A* = total number of examined plants, and *K* = the highest degree of infection.

### 2.5. Estimation of Mycorrhizal Colonization

For each applied treatment, four sunflower roots were evaluated for the level of mycorrhizal colonization with *R. irregularis* at 28 dpi. Small segments (1 cm) of each root were prepared and treated with trypan blue stain, as stated by Phillips and Hayman [25] (Sigma-Aldrich, Saint Louis, MO, USA). Mycorrhizal colonization was estimated in forty root segments of each treatment while using a light microscope as reported by Trouvelot et al. [26].

### 2.6. Biochemical Estimations

Twenty-eight days after inoculation with *R. solani*, the total phenol content was estimated in the sunflower roots according to the method that was described by Malik and Singh [27]. The activities of two defense-related enzymes were also determined. The extraction and assay of polyphenol oxidase (PPO) enzyme were performed according to Galeazzi et al. [28], while, extraction and assay of peroxidase (POD) enzyme were carried out according to Maxwell and Bateman [29].

### 2.7. Statistical Analyses

Statistical significances were analyzed while using the software CoStat (version 6.4). Comparisons between the means were performed using Tukey’s HSD (honestly significant difference) test at *p* ≤ 0.05 [30]. Line plots and hierarchical clustering analysis were performed while using BioVinci Software (Bioturing, San Diego, CA, USA). The line plots were drawn by the default setting of the program, while the clustering heat maps were conducted using the clustering method (ward minimum values), distance method (euclidean) clustered by (column and row).

## 3. Results

### 3.1. Time-Course Changes in the Systemic Transcript Levels of the Polyphenol Biosynthesis-Related Genes

**Figure 1:**
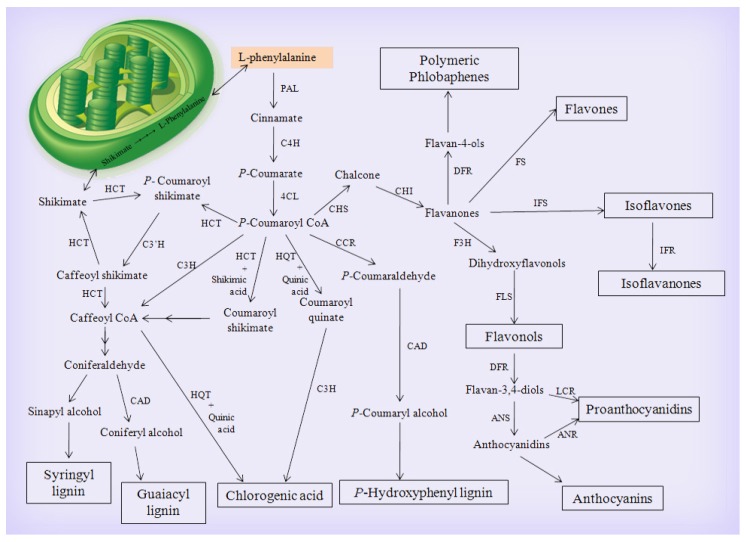
Graphical diagram of the polyphenol biosynthetic pathway (adapted from André et al. [20], Mahesh et al. [21], Albert et al. [22]; Reproduced with permission from [20,21,22]), where, *PAL*: phenylalanine ammonia-lyase, *C4H*: cinnamic acid 4-hydroxylase, *4CL*: 4-coumarate-CoA ligase, *HCT*: hydroxycinnamoyl CoA shikimate hydroxycinnamoyl transferase, *CAD*: cinnamyl alcohol dehydrogenase, HQT: hydroxycinnamoyl CoA quinate hydroxycinnamoyl transferase, *CHS*: chalcone synthase, *CCR*: cinnamoyl-CoA reductase, *CHI*: chalcone isomerase, *DFR*: dihydroflavonol 4-reductase, *FS*: flavone synthase, *C3H*: *p*-coumarate 3-hydroxylase, *F3H*: flavanone 3-hydroxylase, *IFS*: isoflavone synthase, *F3′H*: flavonoid 3′ hydroxylase, *IFR*: isoflavone reductase, *FLS*: flavonol synthase, *LCR*: leucocyanidin reductase, and *ANS*: anthocyanidin synthase.

Changes over time in the systemic transcriptional expression levels of the polyphenol biosynthesis-related genes were monitored in the sunflower leaves at 3, 7, 14, 21, and 28 days-post-inoculation with *R. solani* (dpi) to investigate the cellular responses against root colonization with *R. irregularis* and/or infection with *R. solani*. In addition, the transcriptional expression levels of these genes were also studied in the sunflower root at 28 dpi. The study included 13 genes that were involved in the control of the three divisions of the polyphenol biosynthetic pathway (phenylpropanoid, flavonoid, and chlorogenic acid biosynthetic pathways), as illustrated in Figure 1.

#### 3.1.1. The Main Phenylpropanoid Biosynthetic Pathway

**Figure 2:**
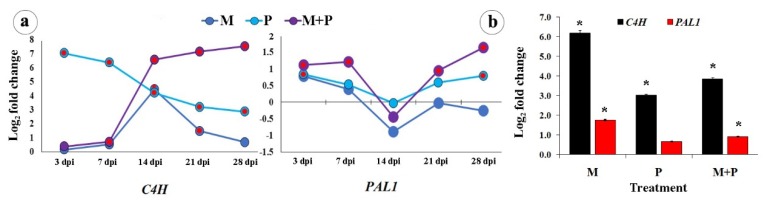
Change in the transcriptional expression levels of the phenylpropanoid biosynthetic pathway genes in sunflower leaves at 3, 7, 14, 21, and 28 dpi (**a**), and roots at 28 dpi (**b**) in response to colonization with *R. irregularis* and/or infection with *R. solani*. Where, M = colonized with *R. irregularis*, P = infected with *R. solani*, and M+P = colonized with *R. irregularis* and infected with *R. solani*. For each gene in the subfigure (**a**), treatments marked with red are significantly different at *p* ≤ 0.05. In the subfigure (**b**), bars indicate the standard error, and for each gene, columns superscripted with asterisks are significantly different at *p* ≤ 0.05.

The changes in the expression of two genes; *PAL1* and *C4H*, were investigated in this pathway. Concerning sunflower leaves, the results that were obtained from qRT-PCR showed that either *R. solani* treatment or dual treatment with *R. irregularis* and *R. solani*, except at 3 dpi, led to considerable up-regulations of the transcriptional expression level of *C4H* higher than *PAL1* gene (Figure 2a). Except at 3 and 28 dpi, the response of the plant toward colonization with *R. irregularis* was significant with concern to *C4H*. In contrast, the infection with *R. solani* mainly provoked the gene expression in the early stages (3 and 7 dpi) with greater change than colonization with *R. irregularis* or the dual treatment. Although the change of *C4H* due to *R. solani* treatment gradually decreased over time, it remained higher than that of *R. irregularis* treatment, except at 14 dpi. While no change was noticed at 3 or 7 dpi, the dual treatment gradually up-regulated the gene expression of *C4H* more than any single treatment at 14, 21, and 28 dpi. On the other hand, the change of *PAL1* showed a relatively lower expression level than *C4H* over the time intervals, while, the significant up-regulation of expression was observed for the dual treatment along the time, except at 14 dpi, and for *R. solani* treatment at 3 and 28 dpi.

In the sunflower root, the results showed that all tested treatments up-regulated the transcriptional expression level of *C4H* more than *PAL1* gene, as observed in the leaves (Figure 2b). Colonization with *R. irregularis* provoked the gene expression of *C4H* or *PAL1* higher than infection with *R. solani* or the dual treatment. The dual treatment came second in this regard, while the *R. solani* treatment was the lowest.

#### 3.1.2. The Flavonoid Biosynthetic Pathway

**Figure 3:**
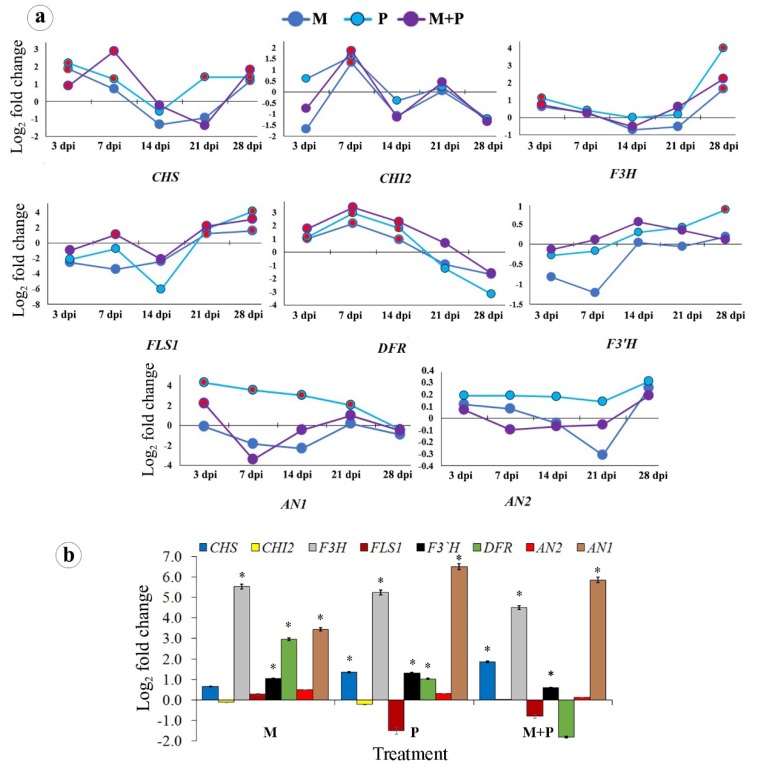
Change in the transcriptional expression levels of the flavonoid biosynthetic pathway genes in sunflower leaves at 3, 7, 14, 21, and 28 dpi (**a**), and roots at 28 dpi (**b**) in response to colonization with *R. irregularis* and/or infection with *R. solani*. Where, M = colonized with *R. irregularis*, P = infected with *R. solani*, and M+P = colonized with *R. irregularis* and infected with *R. solani*. For each gene in the subfigure (**a**), treatments marked with red are significantly different at *p* ≤ 0.05. In the subfigure (**b**), bars indicate the standard error, and for each gene, columns superscripted with asterisks are significantly different at *p* ≤ 0.05.

In this pathway, changes over time in the expression levels of eight genes; *F3H*, *CHS, FLS1, DFR, CHI2, AN2, AN1,* and *F3′H* were studied (Figure 3a,b). In sunflower leaves, change in the gene expression of *CHS, FLS1, DFR,* and *AN1* were observed. For *CHS*, both single treatments of *R. irregularis* or *R. solani* showed a significant change at 3 dpi, which then gradually decreased until 14 dpi, after which a significant up-regulation was only observed for *R. solani* treatment at 21, while, at 28 dpi, all of the treatments showed a significant up-regulation of the gene expression. The dual treatment triggered the gene expression higher than the single treatments at 7 dpi, after which the change gradually decreased until 21 dpi and then the change significantly up-regulated at 28 dpi. For *FLS1*, no significant change was observed for the all of the treatments, while a considerable up-regulation was only noticed at 7 dpi for the dual treatment, followed by sharp down-regulation at 14 dpi. At 21 and 28 dpi, all of the treatments showed significant up-regulation with superiority of *R. solani* treatment. All of the treatments showed significant changes of *DFR* at 3, 7, and 14 dpi. The inducing effect increased, reaching the maximum at 7 dpi, and then gradually decreased over time for all treatments with superiority for the dual treatment. All of the treatments only showed significant up-regulations for *CHI2* gene at 7 dpi. For *F3H* gene, significant changes were observed for all treatments at only 3 and 28 dpi with the superiority of *R. solani* treatment over the other treatments. No changes were observed for *F3′H*, except for *R. solani* treatment at 28 dpi, or *AN2* genes by the single or dual treatments along the different time intervals. At 3 dpi, infection with *R. solani* led to change of *AN1* higher than that of the dual treatment. The significant change of *R. solani* treatment gradually decreased along the time intervals.

In the sunflower root, the highest change was observed for *F3H* and *AN1* genes. In this regard, the obtained results showed that *R. irregularis* treatment up-regulated *F3H* gene higher than *R. solani* treatment, followed by the dual treatment; while, the highest expression of *AN1* was noticed for *R. solani* treatment, followed by the dual treatment, while the *R. irregularis* treatment came third in this concern. For the *DFR* gene, higher expression was observed for the *R. irregularis* treatment, followed by *R. solani* treatment. The treatments of *R. solani* or the dual treatment up-regulated the *CHS* expression, but the dual treatment was more of an inducer than the single treatments. Significant changes of *F3′H* gene expression were noticed for the *R. solani* treatment than the *R. irregularis* treatment. No change for *AN2* and *CHI2* genes was observed for all of the treatments. A down-regulation of the *FLS1* gene was observed for the *R. solani* treatment and the dual treatment, while no change was observed for *R. irregularis* treatment.

#### 3.1.3. The Chlorogenic Acid Biosynthetic Pathway

**Figure 4:**
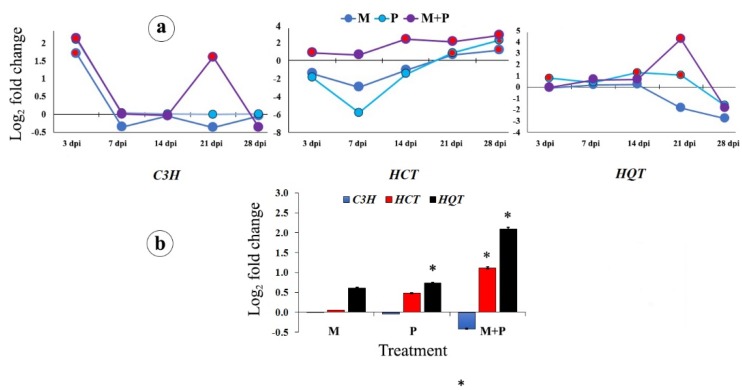
Change in the transcriptional expression of the chlorogenic acid biosynthetic pathway genes in sunflower leaves at 3, 7, 14, 21, and 28 dpi (**a**), and roots at 28 dpi (**b**) in response to colonization with *R. irregularis* and/or infection with *R. solani*. Where, M = colonized with *R. irregularis*, P = infected with *R. solani*, and M+P = colonized with *R. irregularis* and infected with *R. solani*. For each gene in the subfigure (**a**), treatments marked with red are significantly different at *p* ≤ 0.05. In the subfigure (**b**), bars indicate the standard error, and for each gene, columns superscripted with asterisks are significantly different at *p* ≤ 0.05.

Three genes regulating the chlorogenic acid biosynthetic pathway were addressed in this study: *HQT*, *HCT*, and *C3H* (Figure 4a,b). In sunflower leaves, significant up-regulations of the *HCT* gene due to the dual treatment were observed along the studied time, except at 7 dpi, which gradually increased from 14 dpi and remained stable at 21, and then increased at 28 dpi, while, a down-regulation for this gene was observed for both single treatments. At 21 and 28 dpi, the gene expression was up-regulated by all of the tested treatments. For *HQT* gene, significant changes were observed for *R. solani* treatment at 3, 14, and 21 dpi, while no significant changes for the other treatments were observed, except for the dual treatment at 21 dpi, which was more than *R. solani* treatment. All of the treatments led to a down-regulation of the gene expression at 28 dpi. A considerable change of *C3H* expression was observed for the three treatments at 3 dpi, which gradually decreased and disappeared along the rest time interval, except for the dual treatment at 21 dpi.

In the sunflower roots, the dual treatment led to significant changes in the *HQT* and *HCT* gene expression higher than the single treatments, while no change in *C3H* expression was observed for the single treatments, while the dual treatment down-regulated the gene expression.

### 3.2. Hierarchical Clustering Analysis

**Figure 5:**
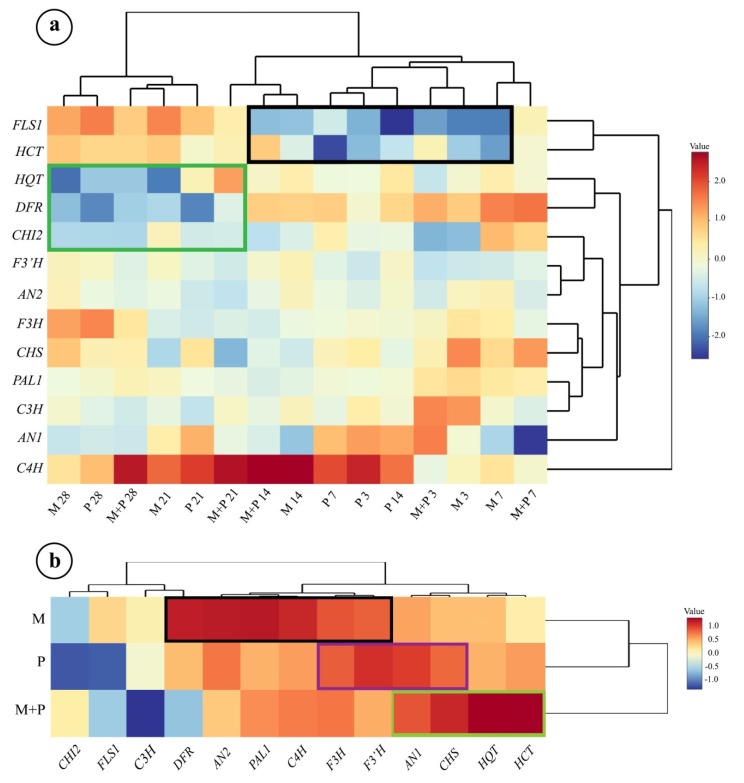
Hierarchical clustering heat maps of change in the transcriptional expression of the polyphenol biosynthetic pathways genes in sunflower leaves at 3, 7, 14, 21, and 28 dpi (**a**), and roots at 28 dpi (**b**) in response to colonization with *R. irregularis* and/or infection with *R. solani*. Where, M = colonized with *R. irregularis*, P = infected with *R. solani*, and M+P = colonized with *R. irregularis* and infected with *R. solani*.

Figure 5a, b represent the hierarchical clustering heat maps of gene expression level in sunflower leaves and roots. With regard to the sunflower leaves, all of the applied treatments along the studied time period are clustered in two main groups, one of them represents the early stages of the study, and the other represents the late stages. In the first group, the single treatment of *R. irregularis* and the dual treatment, at the early stages (3, 7, and 14 dpi), are clustered together in separate subgroups, while the single treatment of *R. solani* at these stages are clustered together in one subgroup.

In the second leading group, treatments of *R. irregularis* and/or *R. solani* at late stages (21 and 28 dpi) are clustered in one group (Figure 5a). Regarding the expression level in each pathway, it was found that *C4H* represented the master expressed gene with the highest collective expression level in the phenylpropanoid biosynthetic pathway. While in the flavonoid biosynthetic pathway, the *AN1* was the highly expressed up-regulated gene. In the chlorogenic acid biosynthetic pathway, *C3H* exhibited the highest change for all of the tested treatments over the time intervals. With regard to the level of gene clustering, the *C4H* gene is clustered in a single out-group which reveals its unique expression level especially in the case of the dual treatment at 14, 21, and 28 dpi. Two-genes-clustering was observed between *PAL1*-*C3H*, *DFR*-*FLS1*, and *HQT*-*HCT*. The hierarchical clustering expression in the early stages (3, 7, and 14 dpi) exhibited down-regulation of *FLS* and *HCT* genes in the case of all treatments (black rectangle). While, in the late stages of all tested treatments (21, and 28 dpi), down-regulations in the gene expressions were observed for *HQT*, *DFR*, and *CHI2* (green rectangle).

The hierarchical clustering heat map of gene expression level in sunflower roots showed that the dual treatment achieved the highest pattern of gene up-regulation (Figure 5b). In this regard, up-regulation was observed for *AN1*, *CHS*, *HQT,* and *HCT,* respectively (green rectangle). However, two clustering groups of gene expression were also observed: *HCT*-*HQT* in one group and *CHS*-*AN1* in the other one. The single treatments of *R. irregularis* and *R. solani* were clustered in one group away from the dual treatment. The main target up-regulated genes by the *R. irregularis* treatment were *DFR, AN2, PAL1, C4H, F3H*, and *F3′H* (black rectangle). Otherwise, *F3H,* *F3′H, AN1,* and *CHS* were the main target that was up-regulated genes by the *R. solani* treatment (violet rectangle).

### 3.3. Plant Growth Evaluation

The results obtained from the greenhouse experiment showed that infection with *R. solani* negatively affects the sunflower plants. Significant reductions in the shoot and root lengths, and dry weights, as well as leaf areas were observed, while the number of leaves did not exhibit any significant difference when compared with the control plants (Table 2). In contrast, sunflower plants colonized with *R. irregularis* whether infected/or not with *R. solani* showed significant improvements in these parameters, except number of leaves, recording the highest values in this regard when compared with the control treatment.

### 3.4. Disease Assessment

**Figure 6:**
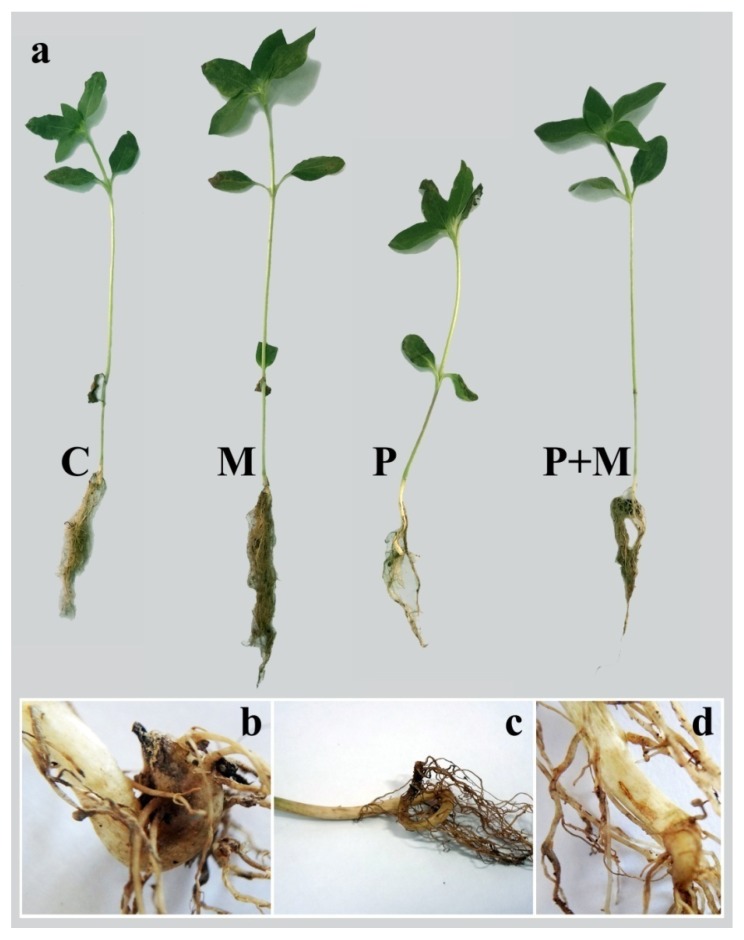
A photograph showing full sunflower plants (at 28 dpi) in response to colonization with *R. irregularis* and/or infection with *R. solani* (**a**), as well as the disease symptoms (**b**–**d**), where, C = untreated control, M = colonized with *R. irregularis*, P = infected with *R. solani*, and M+P = colonized with *R. irregularis* and infected with *R. solani*.

The data of the disease assessment from the greenhouse experiment showed high disease incidence and severity of sunflower plants that were infected with *R. solani*, recording 96.7 and 58.3%, respectively (Table 3). Reddish-brown discoloration, morphological malformations, and necrotic lesions were observed in the roots, as well as the collar region of the stem in addition to stunting, weakness, and wilting in the shoot system (Figure 6). However, colonization with *R. irregularis* significantly mitigated the adverse effects of the disease, achieving 55.1 and 48.5% reduction in the disease incidence and severity, respectively, in comparison with the non-colonized and infected plants. No disease incidence was observed on the non-infected plants.

### 3.5. Estimation of Colonization Level

**Figure 7:**
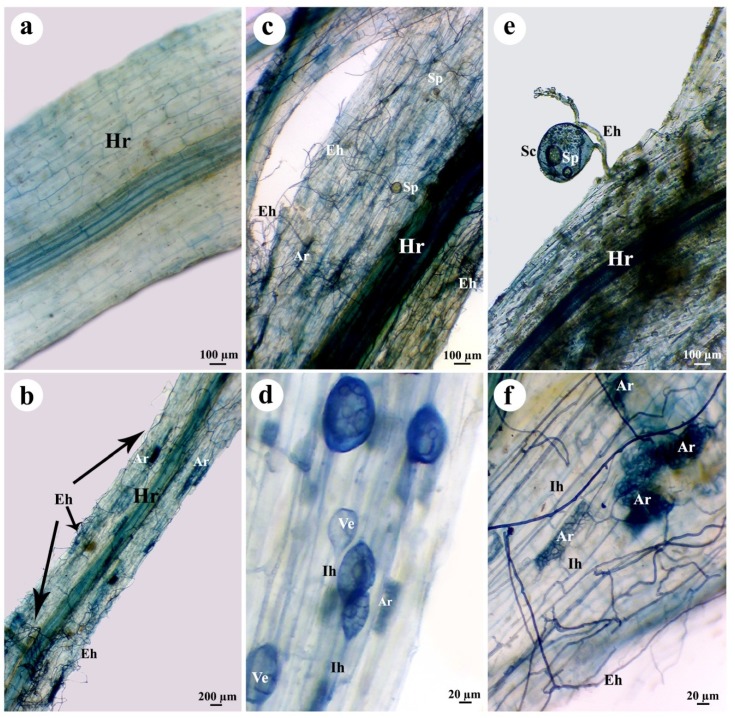
Light micrographs of sunflower roots colonized with *R. irregularis* displaying typical mycorrhizal structures (at 28 dpi), control plant (**a**), and *R. irregularis*-colonized plant (**b**–**f**), where, Hr = host root, Ih = interaradical hyphae, Eh = exteraradical hyphae, Sp = spore, Sc = sporocarp, Ve = vesicle, and Ar = arbuscule.

The roots colonization levels of sunflower plants in response to the applied treatments are presented in Table 4. Sunflower plants treated only with *R. irregularis* showed a high level of mycorrhizal colonization, recording 96.7, 45.3, and 23.4% of colonization intensity and arbuscules frequency, respectively. Light microscopic examination showed typical mycorrhizal structures in the sunflower roots that were treated with *R. irregularis* (Figure 7). However, infection with *R. solani* significantly reduced the colonization level in the sunflower plants that were treated with *R. irregularis* when compared with the non-infected-*R. irregularis* treated plants. No mycorrhizal colonization was noticed in the plants that were non-treated with *R. irregularis*.

### 3.6. Total Phenol Content and Activities of Defense-Related Enzymes

Table 5 presents the means of the total phenol content and activities of polyphenol oxidase (PPO) and peroxidase (POD) enzymes in sunflower roots in response to colonization with *R. irregularis* and/or infection with *R. solani*. The colonization of sunflower roots with *R. irregularis* and/or infection with *R. solani* significantly induced the total phenol content and activities of PPO and POD when compared with the control plants. However, the dual treatment (M+P) achieved the highest values in this regard. Except for POD, infection with *R. solani* (P) led to significant increases in the estimated biochemical measures that were higher than that produced by the colonization with *R. irregularis* (M) when compared to the control treatment.

## 4. Discussion

In this study, two soil-borne fungi were addressed; both of them can penetrate and inhabit the plant roots, one behaves as a friend and the other as a foe. Polyphenolic compounds are plant secondary metabolites that play vital roles in plant growth, development, pigmentation, and resistance against different biotic and abiotic stresses [31,32]. Among these polyphenolic compounds, flavonoids and chlorogenic acid, which constitute the majority of polyphenols, have shown wide antifungal activities against several fungal phytopathogens [16,17]. Time-course changes in the genes expression of chlorogenic acid and flavonoid biosynthetic pathways were investigated in sunflower plant in response to colonization with *R. irregularis* and/or infection with *R. solani*. The results obtained from the hierarchical clustering analysis in this study demonstrated that *C4H*, the key gene of lignin biosynthesis, was the master expressed gene among the addressed genes with the highest collective expression level over the studied time period, which suggests the probable main plant reaction in response to the tested treatments. *C4H* catalyzes the conversion of *trans*-cinnamic acid to *p*-coumaric acid in the phenylpropanoid pathway [33]. Yan et al. [34] reported that the up-regulation of *GmC4H1* in *Nicotiana benthamiana* enhanced the lignin accumulation and triggered the plant resistance against *Phytophthora parasitica* and *Verticillium dahliae*. Moreover, the gene silencing of *GmC4H1* in soybean repressed the plant resistance. The up-regulation of the transcriptional expression levels of *PAL1*, *HCT,* and *C3H* was also observed in this study. *PAL1* catalyzes the first step in the main phenylpropanoid pathway, the conversion of phenylalanine to *trans*-cinnamic acid, providing precursors for all of the emerged metabolites, including flavonoids and lignins [35]. The gene repression of *PAL* and *C4H* in *Arabidopsis* and *Populus* led to considerable reductions in the lignin content [36]. *HCT* and *C3H* are also involved in the early steps of monolignols biosynthesis (sinapyl alcohol and coniferyl alcohol), the monomers of lignin polymer, which utilized in the cell wall lignification [37]. Shinya et al. [38] investigated the correlation between the lignin content and the transcript levels of the lignin formation-related genes in two genotypes of hybrid *Eucalyptus* (AM063 and AM380) while using RNA-seq with total RNAs. They found a correlation between the higher lignin content in AM380 genotype with the highly transcript levels of the common phenylpropanoid pathway genes *PAL, C4H,* and *4CL*. The induction of lignin deposition in the host cell wall due to fungal pathogens has been extensively reported in the literature [39,40]. Cell wall lignification is one of the most effective resistance mechanisms in the plants. It acts as a physical barrier preventing infection and extension of the pathogen in the plant tissue, diffusion of their toxins and hydrolytic enzymes into the plant tissue, and water and nutrients translocation from the plant to the pathogen [41]. The elicitation of lignin accumulation due to mycorrhizal colonization has also been reported. Abdel-Fattah et al. [15] reported cell wall thickening in the roots of mycorrhizal bean plants due to lignin deposition against infection with *R. solani*. Triggering cell wall lignification due to root mycorrhizal colonization enhances plant resistance against invading pathogens. The obtained results in this study regarding the overexpression of the lignification-related genes are in agreement with that from previous investigations. However, the plant response toward colonization with *R. irregularis* was higher than that due to infection with *R. solani*, particularly in the roots.

On the other hand, the data from this study showed an up-regulation of *FLS1* at the late stages of the time intervals for all treatments with the superiority for *R. solani* treatment, while the up-regulation of *DFR* and *AN1* was observed at the early stages for all of the tested treatments with superiority for the dual treatment. This result is in agreement with the findings obtained from other investigations that reported the disequilibrium of *FLS1* and *DFR* expression patterns [42]. This result can be discussed in the light of the substrate competition between *FLS1* and *DFR*. The two enzymes compete for the same substrate (dihydroflavonols), where *FLS1* catalyzes the conversion of dihydroflavonols to produce a set of flavonols [43], while *DFR* catalyzes their conversion to form leucoanthocyanidins in the anthocyanins-biosynthetic pathway [44]. Accordingly, the up-regulation of any gene toward one route might negatively affect the other route. In this regard, it was found that the overexpression of the *FLS* genes in tobacco led to the accumulation of flavonols and inhibition of anthocyanin biosynthesis, while the overexpression of *DFR* genes in tobacco triggered anthocyanin accumulation [42,45]. The obtained results in this study showed an up-regulation of *AN1* at the early stages in the sunflower leaves, while their overexpression was observed in the roots at the late stage. *AN1* is a regulatory gene that is involved in the anthocyanins biosynthesis [46]. Quattrocchio et al. [47] demonstrated that in petunia and maize the genes *AN1*, *AN2*, *AN4*, and *AN11* regulate the transcription of a subset of structural genes from the anthocyanin pathway by using a combination of RNA gel blot analysis and transcription run-on assays confirming their role in pigmentation in leaf cells. Anthocyanins are flavonoid pigments that naturally accumulated in the plants in response to light, stresses, and different inducers. In plant, the anthocyanins have many physiological roles, including defense against biotic stress [48]. However, their function might vary according to the plant tissue; the leaf’s anthocyanins may act as photoprotectant or visual signals, while, in the roots, they are more likely to act as a toxin [49]. In this regard, several investigations reported the antifungal activity of anthocyanins against various fungi [48,50,51].

Among the results obtained in this study is the overexpression of *CHS, CHI,* and *F3H* genes by the three tested treatments in sunflower leaves and roots at various stages of the addressed time intervals. *CHS* catalyzes the condensation reaction of *p*-coumaroyl-CoA to form naringenin chalcone, which is isomerised to flavanone by *CHI* and then converted to dihydroflavonols by *F3H,* from which different types of flavonoids are formed in the flavonoid biosynthetic pathway. Bovy and his team [52] studied the flavonoid pathway in *Solanum lycopersicum* by increasing endogenous flavonoids and blocking flavonoid pathway by RNA interference. The metabolite profiling revealed that the *CHS*, *CHI*, and *F3H* genes not only affect the flavonoid composition itself, but also on the other related or unrelated metabolic pathways. The overexpression of these genes might lead to the high accumulation of flavonoid and isoflavonoid phytoalexins and phytoanticipins, which play important roles in plant immunity against phytopathogens [53]. Phytoanticipins and phytoalexins are both low-molecular-weight, antimicrobial secondary metabolites that formed from different origins, including flavonoids, but phytoalexins, are accumulated in the plant in response to pathogenic attack, while phytoanticipins are naturally produced in plants without the need for pathogenic challenge [54,55]. Moreover, phytoalexins have been reported to be accumulated in response to mycorrhizal colonization enhancing the plant resistance against pathogenic invasions [56]. This explanation is supported by the enhanced phenolic contents in sunflower roots that were noticed in this study, which are also following the results obtained in other studies.

One of the overexpressed genes in this study is *HQT,* which was up-regulated, particularly in the roots, at the late stage by all treatments with the superiority for the dual treatment over the single treatments pointing out that chlorogenic acid accumulation seem to be a probable plant reaction toward the applied treatments. *HQT* is a key gene in the chlorogenic acid biosynthesis that catalyzes the conversion of caffeoyl-Co A to form chlorogenic acid [57]. In this regard, Payyavula et al. [58] found that *HQT*-silencing led to a high reduction in the chlorogenic acid production in potato. Chlorogenic acid is one of the most important defensive phenolic compounds that are produced in the plant in response to pathogen attack [17]. It is a versatile defense secondary metabolite. It has a potent antimicrobial activity when it oxidized via polyphenoloxidases to form the corresponding quinone, chlorogenoquinone, which can inactivate pathogenic enzymes [59]. The overexpression of *HQT* in the sunflower roots, the site of infection, in response to colonization with *R. irregularis* and/or infection with *R. solani* is supported by the induced activities of PPO enzyme in sunflower roots that were observed in this study. Moreover, it has the ability to interfere with the infection-related fungal processes, such as toxin production and appressorium formation, rather than the fungal growth [60].

Hierarchical clustering analysis in this study showed two-genes-clustering in different biosynthetic pathways groups, such as *DFR*-*FLS* and *HQT*-*HCT*, which reveals that these genes seem to be coordinately transcribed and indicates the coherence in these pathways. The clustering of *HQT* and *HCT* in one group is logical, being the key genes in the chlorogenic acid biosynthetic pathway. The same pattern of clustering was seen for *F3H* and *CHS,* the key genes in the biosynthesis of different flavonoids. On the other hand, clustering between the applied treatments along the studied time intervals was also noticed in this study. In sunflower leaves, the clustering of the single treatment of *R. irregularis* with the dual treatment, particularly in the early stages of the study indicates that *R. irregularis* is likely to have an overriding effect over *R. solani* in the dual treatment at these stages, while taking the temporal precedence of colonization with *R. irregularis* over the infection with *R. solani* in account. While the clustering of all tested treatments at the late stages in one main group reveals the coordination and similarity between their inducing behaves at the late stages than the early ones. Indeed, a successful mycorrhizal relationship requires intense coordination between the fungus and the plant host. Upon mycorrhizal establishment, a transcriptional reprogramming takes place in the plant, which results in a set of modulations in the plant metabolism [61], mostly secondary metabolites biosynthesis, and it leads to triggering their innate and adaptive immune responses [62,63]. The degree of modulations varies according to the host, fungus, and the developmental phase of colonization. These induced defense responses correlate with the activation of jasmonic acid-dependent signaling pathway, and the repression of salicylic acid-dependent signaling pathway [64]. However, regulatory interactions between both hormones signaling pathways also occur [65]. As a result of this earlier priming, the plant gets systemically more resistant against pathogen attack [66].

On the other hand, improving plant growth by mycorrhizal association is a well-known result, owing to the much beneficial facilitation provided by the mycorrhizal fungus. Enhancement of sunflower plant growth reported in this study due to association with *R. irregularis* is in accordance with that obtained by Aseel et al. [67] on tomato plants. In the mycorrhizal relationship, it is widely accepted that mycorrhizal fungus provides the plant with water and nutrients from soil via their highly branched extra-radical mycelial network that might interconnect many adjacent plants in the same site with each other [68]. In addition to their enhanced water and nutrient uptake that may help in the damage compensation, the mycorrhizal fungus possesses enzymatic activity, which enables it to change availability of the soil nutrients promoting the acquisition of mineral nutrients by the plant [69]. Moreover, it has the ability to produce growth regulators via their intra-radical arbuscules improving the plant photosynthesis and metabolism [70]. These mycorrhizal benefits, as well as triggering the plant resistance, can explain the ameliorating impact of *R. irregularis* on the disease damages that resulted from *R. solani* in the dual treatment.

## 5. Conclusions

In conclusion, this study demonstrated that both tested fungi systemically triggered the transcriptional expression level of most of the addressed flavonoid and chlorogenic acid biosynthetic pathways genes at varied degrees. However, the triggering effect differs according to the treatment, gene, and time stage. The inducing effect of *R. irregularis* was more prevalent than that of *R. solani* in the early stages. At the late stages, the inducing effect of all treatments was higher than at the early stages. In general, the dual treatment showed a superior inducing effect over the single treatments at most of the time. Hierarchical clustering analysis showed that *C4H* was the master expressed gene among the addressed genes along the studied time period. However, gene expression changes do not necessarily imply changes in gene product or enzyme activity related to the gene. Accordingly, the most probable plant-defensive-mechanism systemically induced in response to the two tested fungi seems to be the cell wall lignification. In addition, the accumulations of chlorogenic acid, flavonoids, and anthocyanins may be also triggered. Moreover, colonization with *R. irregularis* improved the plant growth, reduced the disease severity that resulted from the infection with *R. solani*, and enhanced the plant immunity in the dual treatment. We can conclude that the proactive, rather than curative, colonization of sunflower plants with *R. irregularis* is of great importance owing to their protective and growth-promoting roles; even if there is no infection when *R. solani* occurred.

## Figures and Tables

**Table 1:** Primer sequences of flavonoid and chlorogenic acid biosynthetic pathways genes.

Primer Name	Abbreviation		Sequence (5’-3’)
*β*-actine	*β*-actine	F	GTGGGCCGCTCTAGGCACCAA
		R	CTCTTTGATGTCACGCACGATTTC
Phenylalanine ammonia-lyase 1	*PAL1*	F	ACGGGTTGCCATCTAATCTGACA
		R	CGAGCAATAAGAAGCCATCGCAAT
Cinnamic acid 4-hydroxylase	*C4H*	F	CCCAGTTTTTGGAAATTGGCTTCA
		R	GCCCCATTCTAAGCAAGAGAACATC
Chalcone synthase	*CHS*	F	CACCGTGGAGGAGTATCGTAAGGC
		R	TGATCAACACAGTTGGAAGGCG
Chalcone isomerase 2	*CHI2*	F	GGCAGGCCATTGAAAAGTTCC
		R	CTAATCGTCAATGATCCAAGCGG
Flavanone 3-hydroxylase	*F3H*	F	CCAAGGCATGTGTGGATATGGACC
		R	CCTGGATCAGTATGTCGTTCAGCC
Flavonol synthase 1	*FLS1*	F	CCTCCTTCCTACAGGGAAGCAAA
		R	CAAGCCCAAGTGACAAGCTCCTAA
Dihydroflavonol 4-reductase	*DFR*	F	TCACAGGAGCAGCTGGATTTATCG
		R	TCAGGATCACGAACAGTAGCATGG
Flavonoid 3′ hydroxylase	*F3′H*	F	TGGGTATACCCAAACTCATTCCG
		R	AAAAGCCCAAAGTTGATGTGAAAGG
Anthocyanin 1 transcription factor	*AN1*	F	CCTCAACCTCAGAAATTCAGAAGC
		R	TCGTTGTTGTTGTCGTTCGATGC
Anthocyanin 2 transcription factor	*AN2*	F	ACAAGATGCCACTTTCCTTCACC
		R	TGTGCATCGTTGGGAGTTAGG
Hydroxycinnamoyl Co A shikimate hydroxycinnamoyl transferase	*HCT*	F	TCTCCAACCCCTTTTAACGAACC
		R	CAACTTGTCCTTCTACCACAGGGAA
Hydroxycinnamoyl Co A quinate hydroxycinnamoyl transferase	*HQT*	F	CCCAATGGCTGGAAGATTAGCTA
		R	CATGAATCACTTTCAGCCTCAACAA
*p*-coumarate 3-hydroxylase	*C3H*	F	TTGGTGGCTACGACATTCCTAAGG
		R	GGTCTGAACTCCAATGGGTTATTCC

**Table 2:** Growth parameters of sunflower plants in response to colonization with *R. irregularis* and/or infection with *R. solani* (28 days after inoculation with *R. solani*).

Treatment *	Shoot Length (cm)	Root Length (cm)	Shoot Dry Weight (g)	Root Dry Weight (g)	No. of Leaves	Leaf Area (cm^2^)
C	26.3 ± 0.6 ^b^	16.7 ± 0.5 ^b^	0.42 ± 0.03 ^b^	0.26 ± 0.02 ^b^	7.0 ± 0.2 ^a^	12.81 ± 0.3 ^b^
M	33.0 ± 1.1 ^a^	19.3 ± 0.7 ^a^	0.52 ± 0.09 ^a^	0.34 ± 0.06 ^a^	7.6 ± 0.4 ^a^	17.07 ± 0.2 ^a^
P	21.7 ± 1.0 ^c^	11.3 ± 0.9 ^c^	0.32 ± 0.02 ^c^	0.15 ± 0.09 ^c^	6.0 ± 0.3 ^a^	9.24 ± 0.2 ^c^
M+P	35.3 ± 1.0 ^a^	18.7 ± 0.3 ^a^	0.55 ± 0.05 ^a^	0.31 ± 0.08 ^a^	7.6 ± 0.5 ^a^	16.93 ± 0.1 ^a^

C = untreated control, M = colonized with *R. irregularis*, P = infected with *R. solani*, M+P = colonized with *R. irregularis* and infected with *R. solani.* * Values of each column followed by the same letter are not significantly different according to Tukey’s HSD test (*p* ≤ 0.05), each value represents the mean of four replicates ± SD.

**Table 3:** Disease assessment of sunflower plants in response to infection with *R. solani* and/or colonization with *R. irregularis* (28 days after inoculation with *R. solani*).

Treatment *	Disease Incidence (%)	Disease Severity (%) **
C	0 ^c^	0 ^c^
M	0 ^c^	0 ^c^
P	96.7 ± 3.7 ^a^	58.3 ± 2.8 ^a^
M+P	53.3 ± 3.5 ^b^	28.3 ± 2.7 ^b^

C = untreated control, M = colonized with *R. irregularis*, P = infected with *R. solani*, and M+P = colonized with *R. irregularis* and infected with *R. solani.* * Values of each column followed by the same letter are not significantly different according to Tukey’s HSD test (*p* ≤ 0.05), each value represents the mean of four replicates ± SD. ** Disease severity was estimated according to Carling et al. [24].

**Table 4:** Levels of mycorrhizal colonization in sunflower plants treated with *R. irregularis* in response to the infection with *R. solani* (28 days after inoculation with *R. solani*).

Treatment *	F (%)	I (%)	A (%)
C	0 ^c^	0 ^c^	0 ^c^
M	96.7 ± 1.1 ^a^	45.25 ± 1.0 ^a^	23.4 ± 0.9 ^a^
P	0 ^c^	0 ^c^	0 ^c^
M+P	90.5 ± 0.9 ^b^	14.33 ± 0.8 ^b^	7.5 ± 1.0 ^b^

C = untreated control, M = colonized with *R. irregularis*, P = infected with *R. solani*, M+P = colonized with *R. irregularis* and infected with *R. solani*, F% = frequency of root colonization, I% = intensity of cortical colonization, and A% = arbuscules frequency. * Values of each column followed by the same letter are not significantly different according to Tukey’s HSD test (*p* ≤ 0.05), each value represents the mean of four replicates ± SD.

**Table 5:** The mean total phenol content and activities of polyphenol oxidase (PPO) and peroxidase (POD) enzymes in sunflower roots in response to colonization with *R. irregularis* and/or infection with *R. solani* (28 days after inoculation with *R. solani*).

Treatment *	Total Phenol Content (mg g^−1^ Fresh Weight)	PPO (U mL^−1^ min^−1^)	POD (U mL^−1^ min^−1^)
C	1.813 ± 0.095 ^d^	0.213 ± 0.033 ^d^	0.153 ± 0.086 ^c^
M	2.129 ± 0.046 ^c^	0.334 ± 0.045 ^c^	0.210 ± 0.061 ^b^
P	2.499 ± 0.064 ^b^	0.455 ± 0.015 ^b^	0.221 ± 0.048 ^b^
M+P	2.873 ± 0.191 ^a^	0.554 ± 0.012 ^a^	0.307 ± 0.027 ^a^

C = untreated control, M = colonized with *R. irregularis*, P = infected with *R. solani*, M+P = colonized with *R. irregularis* and infected with *R. solani.* * Values of each column followed by the same letter are not significantly different according to Tukey’s HSD test (*p* ≤ 0.05), each value represents the mean of four replicates ± SD.
